# Heart Failure Severity Closely Correlates with Intestinal Dysbiosis and Subsequent Metabolomic Alterations

**DOI:** 10.3390/biomedicines10040809

**Published:** 2022-03-30

**Authors:** Martina E. Spehlmann, Ashraf Y. Rangrez, Dhiraj P. Dhotre, Nesrin Schmiedel, Nikita Chavan, Corinna Bang, Oliver J. Müller, Yogesh S. Shouche, Andre Franke, Derk Frank, Norbert Frey

**Affiliations:** 1 Department of Internal Medicine III, Cardiology, Angiology and Intensive Care Medicine, University Hospital of Schleswig-Holstein, Rosalind-Franklin Str. 12, 24105 Kiel, Germany; martina.spehlmann@uksh.de (M.E.S.); nesrin.schmiedel@uksh.de (N.S.); oliver.mueller@uksh.de (O.J.M.); derk.frank@uksh.de (D.F.); 2 German Centre for Cardiovascular Research (DZHK), Partner Site Hamburg/Kiel/Lübeck, 24105 Kiel, Germany; 3 Department of Internal Medicine III, University of Heidelberg, Im Neuenheimer Feld 410, 69120 Heidelberg, Germany; 4 German Centre for Cardiovascular Research (DZHK), Partner Site Heidelberg/Mannheim, 69120 Heidelberg, Germany; 5 National Centre for Cell Science, Pune 411021, India; dhiraj.dhotre@nccs.res.in (D.P.D.); chavannikitas94@gmail.com (N.C.); yogesh@nccs.res.in (Y.S.S.); 6 Institute of Clinical Molecular Biology, Christian-Albrechts-University Kiel, Rosalind-Franklin-Strasse 12, 24105 Kiel, Germany; c.bang@ikmb.uni-kiel.de (C.B.); a.franke@ikmb.uni-kiel.de (A.F.)

**Keywords:** heart failure, gut microbiome, gut–heart axis, dysbiosis, circulating metabolites

## Abstract

Growing evidence suggests an altered gut microbiome in patients with heart failure (HF). However, the exact interrelationship between microbiota, HF, and its consequences on the metabolome are still unknown. We thus aimed here to decipher the association between the severity and progression of HF and the gut microbiome composition and circulating metabolites. Using a mouse model of transverse aortic constriction (TAC), gut bacterial diversity was found to be significantly lower in mice as early as day 7 post-TAC compared to Sham controls (*p* = 0.03), with a gradual progressive decrease in alpha-diversity on days 7, 14, and 42 (*p* = 0.014, *p* = 0.0016, *p* = 0.0021) compared to day 0, which coincided with compensated hypertrophy, maladaptive hypertrophy, and overtly failing hearts, respectively. Strikingly, segregated analysis based on the severity of the cardiac dysfunction (EF < 40% vs. EF 40–55%) manifested marked differences in the abundance and the grouping of several taxa. Multivariate analysis of plasma metabolites and bacterial diversity produced a strong correlation of metabolic alterations, such as reduced short-chain fatty acids and an increase in primary bile acids, with a differential abundance of distinct bacteria in HF. In conclusion, we showed that HF begets HF, likely via a vicious cycle of an altered microbiome and metabolic products.

## 1. Introduction

The microbiota, which is an “ecological community of commensal, symbiotic and pathogenic microorganisms” [1,2], is gaining increasing attention due to its modulating function in several human diseases. Gut dysbiosis, i.e., changes in the physiological composition of gut microbiota due to disease conditions, has increasingly been associated with several cardio-metabolic diseases [3]. Bacterial colonization and translocation of their toxins to the bloodstream due to altered intestinal permeability are directly correlated with systemic inflammation [4]. In this regard, the activation of proinflammatory pathways and chronic inflammation was hypothesized as a major contributing factor in the pathogenicity and progression of heart failure (HF), which is a multisystem disorder and a leading cause of mortality and morbidity worldwide [5,6]. Hence it is thought that the changes in gut microbial content and/or its metabolic products likely affect the heart function and vice versa. In our pilot study, we provided evidence that patients with HF reveal dysbiosis of intestinal microbiota compared to healthy individuals [7,8,9,10,11]. Along these lines, Beale et al. [12] and the authors from the GUMPTION study [13] demonstrated key gut microbial changes in patients with heart failure with preserved ejection fraction (HFpEF). Similarly, Sun et al. showed the occurrence of gut dysbiosis in chronic HF patients [14]. Furthermore, an interesting study from Hayashi and colleagues, using whole-genome shotgun sequencing of fecal samples and mass-spectrometry-based profiling of amino acids demonstrated the relationship between gut dysbiosis and amino acid metabolic disturbances in patients with HF [15]. However, it is not yet clear whether the altered gut microbiome observed in HF patients is really an effect, whether these effects are dependent on the severity of the disease, and whether and how an altered gut microbiome affects cardiac homeostasis.

To establish cause and effect, controlled experimental time course models are necessary to understand the mechanisms and the interrelationship between heart disease and the microbiome. In recent years, mouse models have been used to study not only the impact of the intestinal microbiota on host physiology and the onset of gastrointestinal diseases but also to investigate metabolic and neuronal disorders, cancers, and various other diseases [16,17]. The study of HF in laboratory mice bears significant advantages since mice bred in the same vivarium share similar intestinal microbiota and genetics. Thus, identical conditions could be applied within the commonly used models of HF. For example, in a mouse model of autoimmune myocarditis, Barin et al. demonstrated that the depletion of intestinal bacteria by antibiotics can alleviate experimental myocarditis [18]. Similarly, a recent study in an experimental myocarditis mouse model suggested that fecal transplantation can improve myocardial injury by decreased infiltration of inflammatory cells [19]. Along these lines, we employed the mouse model of transverse aortic constriction (TAC) since this model is well established for inducing pressure overload and subsequent cardiac failure [20]. Using a controlled experimental setup of mice, evaluation of gut microbiome at different stages and severity of cardiac dysfunction, and multi-variate association studies of the gut microbiome and circulating metabolites, we specifically aimed to evaluate the role of host–microbiota correlation in the development of HF.

## 2. Materials and Methods

### 2.1. Animals

**Figure 1:**
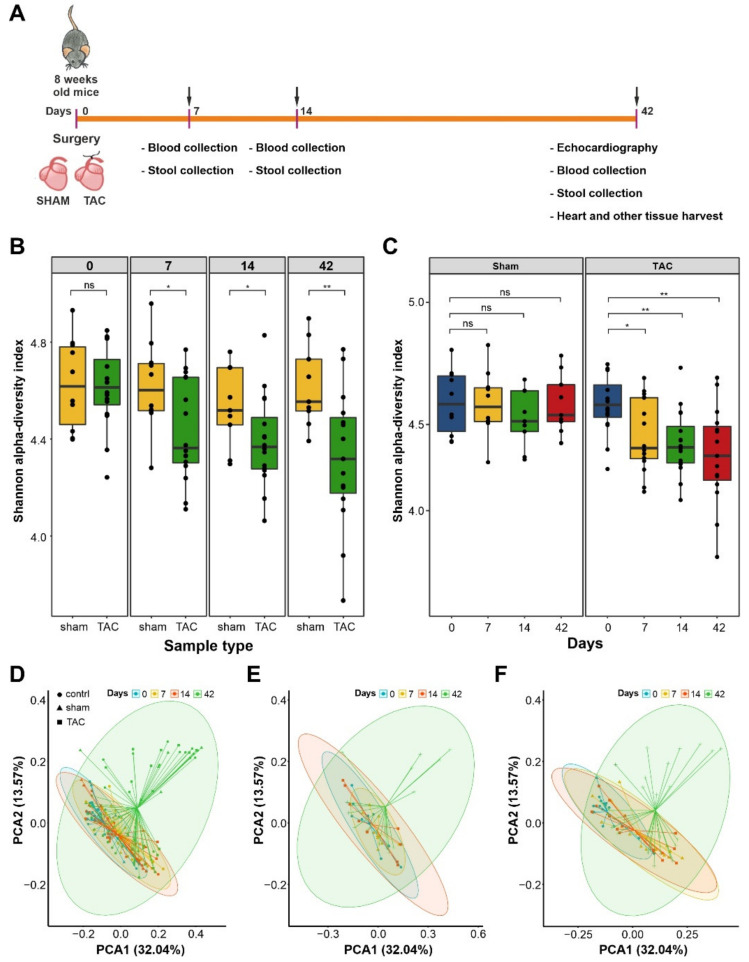
Diversity differences between Sham and TAC sample types. (**A**) Pictorial representation of the study plan. (**B**) Box plots for the Shannon alpha-diversity index in Sham and TAC samples at different time points. The box plot boxes indicate interquartile ranges (IQR), the black line indicates the median, whiskers extend till extreme values within 1.5 times the IQR, and black points outside whiskers are outliers. For the 1st- and 6th-week samples, Shannon index values with significant differences in the Sham and TAC samples are shown (*, **, and ns correspond to *p* ≤ 0.05, *p* ≤ 0.01, and not significant, respectively). (*n* values are as follows: Sham day 0 = 10, day 7 = 10, day 14 = 9, day 42 = 9; TAC day 0 = 16, day 7 = 16, day 14 = 16, day 42 = 15). (**C**) Box plots for the Shannon alpha-diversity index within sample type (Sham and TAC) at different time points. Observed significant differences for each timepoint sample comparison with the 0th-day TAC samples. (*n* values are as follows: Sham day 0 = 10, day 7 = 10, day 14 = 9, day 42 = 9; TAC day 0 = 16, day 7 = 16, day 14 = 16, day 42 = 15). (**D**) For different time points, separate clusters were observed in the PCoA plot for all sample types. For the 6th-week samples, distinct clusters were observed for the control, Sham, and TAC sample types (PERMANOVA *p*-value: 0.001, beta dispersion *p*-value: 1 × 10^−4^). (*n* values are as follows: control day 0 = 12, day 7 = 12, day 14 = 12, day 42 = 12; Sham day 0 = 10, day 7 = 10, day 14 = 9, day 42 = 9; TAC day 0 = 16, day 7 = 16, day 14 = 16, day 42 = 15). (**E**) PCoA plot of Sham samples at different time points. The 6th-week samples formed a separate cluster compared to other timepoint samples for the Sham sample type. (PERMANOVA *p*-value: 0.377, beta dispersion *p*-value: 0.3875) (*n* values are as follows: day 0 = 10, day 7 = 10, day 14 = 9, day 42 = 9). (**F**) PCoA analysis of TAC samples at different time points. The 6th-week samples formed a distinct cluster as compared to other time point data of TAC samples with large variations. (PERMANOVA *p*-value: 0.001, beta dispersion *p*-value: 1 × 10^−4^) (*n* values are as follows: day 0 = 16, day 7 = 16, day 14 = 16, day 42 = 15).

All animal experiments were locally approved by the Ministry of Energy Transition, Agriculture, Environment, Nature, and Digitalization (MELUND) of the state of Schleswig-Holstein (V241-60965/2017 (129-10/17)). The study conformed to the principles outlined in the Declaration of Helsinki and the animal experiments were carried out stringently following the ethical guidelines of the University of Kiel. C57BL/6J mice were purchased at 6 weeks of age from Charles River Laboratories, Sulzfeld, Germany; fed a standard rodent chow diet; and kept under 12 h light and dark cycles. After arrival in our laboratory, the mice were accustomed to the local vivarium for 2 weeks before starting the experiments. The mice were 8-weeks-old at the beginning of the trial (Figure 1A).

### 2.2. Model of Transverse Aortic Constriction

For the TAC experiment, mice were anesthetized with buprenorphine, intubated, and kept under anesthesia using 3% isoflurane, as previously described [20]. Briefly, the body temperature of each mouse was kept stable using a temperature regulation pad. After midline sternotomy, a Prolene 6.0 suture was placed around the aorta between the branching of the brachiocephalic artery and the left carotid artery. A blunt 27-gauge needle was positioned along the aorta and the suture was tightened around the needle. After removing the needle, the chest was closed and the overlying skin was adapted using a Vicryl 5.0 suture. Postoperatively, buprenorphine was administered in the drinking water for 2 days. In mice that underwent the Sham procedure, the same procedures were carried out except for the placement of the suture around the aorta. A total of 39 mice were subjected to the trial. Seventeen mice received a TAC, 10 mice underwent the Sham procedure, and 12 mice were control mice that did not receive treatment of any kind. All mice were kept in individual cages post-surgeries. Stool samples (fresh) were collected on days 0, 7, 14, and 42 of the trial and immediately frozen at −80 °C for storage. All mice were sacrificed on day 42. The hearts and lungs were weighed and immediately frozen in liquid nitrogen. Mouse blood was collected for plasma preparations by cardiac puncture in EDTA-coated (5 mM) tubes centrifuged at 6000× rpm for 10 min at 4 °C and clear plasma was transferred to fresh tubes.

Transthoracic echocardiography was performed on days 14 and 42 using the VisualSonics Vevo 3100 ultrasound system. The heart rate was kept at 400–500 bpm during the procedure. Recordings obtained were in two-dimensional B- and M-modes. Cardiac output (CO), stroke volume (SV), end-diastolic volume (EDV), and end-systolic volume (ESV) were determined based on B-mode images of the parasternal long axis.

### 2.3. DNA Extraction and Sequencing of Bacterial 16S rDNA

We used the QIAamp DNA stool kit (Qiagen) automated on the QIAcube and a prior bead-beating step to extract DNA from the murine stool/ileum samples. The 16S rRNA gene (variable regions v1–v2) was amplified using the primers described by Caporaso et al. [21]. The SequalPrep Normalization Plate Kit (Life Technologies) was used to normalize PCR products. These were pooled (based on the Qubit dsDNA BR Assay Kit measurements) (Thermo Fisher) and sequenced on an Illumina MiSeq (2 × 300 bp). According to the barcode sequences, demultiplexing was based on zero mismatches. The 16S rDNA sequencing data were deposited to the European Nucleotide Archive (ENA, https://www.ebi.ac.uk/ena/browser/home, accessed on 9 August 2021) and are available under the accession number PRJEB45533.

### 2.4. Metabolite Analyses in the TAC Model

For metabolite analyses, plasma samples were analyzed using liquid chromatography coupled with tandem mass spectrometry and gas chromatography–mass spectrometry, as previously described [22,23,24,25]. Briefly, the GC–MS system consisted of an Agilent 6890 GC coupled to an Agilent 5973 MSD (Agilent, Waldbronn, Germany), and the autosamplers were CompiPal or GCPal from CTC (CTC, Zwingen, Switzerland). High-performance liquid chromatography (HPLC) was performed via gradient elution using methanol/water/formic acid on reversed-phase separation columns. Target and high sensitivity MRM (multiple reaction monitoring) profiling in parallel to a full-screen analysis was performed using mass spectrometric detection. MS detection was performed with repetitive cycles of MRM transitions for important preselected metabolites followed by a full scan from *m*/*z* 100 to 1000. The HPLC instruments were purchased from Agilent 1100 (Agilent, Waldbronn, Germany), while the MS instruments were the model API4000 from SCIEX (AB SCIEX, Darmstadt, Germany).

### 2.5. Processing of 16S rRNA Data

16S rRNA sequencing data for 258 samples were analyzed using a DADA2 (v1.13.1) pipeline for identification of amplicon sequence variants (ASVs) (https://benjjneb.github.io/dada2/tutorial.html, accessed on 10 July 2021) [26]. Raw reads were trimmed at 240 nucleotide positions and filtered with a quality cut-off of 20 and an expected error threshold of 2. Further, filtered reads were dereplicated, denoised, and checked for chimeric sequences using default parameters of the DADA2 pipeline. Overall, the sequence table was comprised of 258 samples with an average number of reads per sample of 23,651 ± 6636. Out of 3081 ASVs, 1585 ASVs with an overall abundance of more than 10 were considered for taxonomic assignments using the Ribosomal Database Project (RDP) naive Bayesian classifier [27] with the SILVA (v132) database [28]. Further analysis was carried out in R (v3.6) using the packages phyoseq [29], vegan [30], microbiome [31], metgenomeSeq [32], and ggplot2.

### 2.6. Bacterial Compositional Analysis

To analyze the microbial diversity and richness within different sample types at different time points, Chao1 (richness), Shannon (richness and evenness), and Simpson (evenness) alpha-diversity indices were calculated using the estimate_richness function implemented in the phyloseq R package, and differences within sample types (control, Sham, and TAC) were tested using the non-parametric Wilcoxon’s test. The beta diversity analyses were carried out using generalized UniFrac distances, as implemented in the GUniFrac and ggplot2 R packages, and significant grouping in samples was assessed using permutational multivariate analysis of variance (PERMANOVA). To identify differentially abundant ASV’s within different sample types, linear discriminant analysis effect size (LefSe) was used as implemented on the Galaxy server [33]. To identify the difference in microbial abundances in the two groups, the Wilcoxon test was performed, and the *p*-values were adjusted using the false discovery rate (FDR) method.

### 2.7. Functional Analysis

To predict the metabolic potential of microbial communities from 16S rRNA sequencing data in terms of KEGG orthology (KO), Phylogenetic Investigation of Communities by Reconstruction of Unobserved States (PICRUSt2) was used [34]. Statistical analysis using the Kruskal–Wallis test was done to identify differentially abundant KEGG modules within different sample types. Statistical Analysis of Metagenomic Profiles (STAMP) was used to identify significant pathways that were differentially abundant within different sample types [35]. Normalized metabolome data for the 2nd- and 6th-week TAC/Sham samples were considered for correlation analysis. A total of 81 metabolites that were either differentially abundant (*p* < 0.05, ANOVA test) or those that belonged to important metabolite classes (amino acids, carbohydrates, complex lipids and fatty acids, and energy metabolism) were used to find correlations with 50 differentially abundant genera using Pearson’s correlation. Correlation networks were generated using Cytoscape v3.8.0.

### 2.8. Statistics

All the results are presented as box plots with a minimum-to-maximum range or bar graphs (showing all points) unless specified otherwise. Statistical significance within sample types (control, Sham, and TAC) for bacterial composition analysis was tested using non-parametric Wilcoxon’s test and the significant grouping in samples was assessed using permutational multivariate analysis of variance (PERMANOVA). To identify the difference in microbial abundances between the two groups, Wilcoxon’s test was performed, and the *p*-values were adjusted using the false discovery rate (FDR) method. Mice echocardiography data were analyzed using one-way analysis of variance (ANOVA, followed by the Student–Newman–Keuls post hoc test), whereas statistical analysis using the Kruskal–Wallis test was used to identify differentially abundant KEGG modules within different sample types. The *p*-values  ≤  0.05 were considered statistically significant.

## 3. Results

### 3.1. HF Induced Changes in Bacterial Composition and Loss of Diversity of Fecal Bacteria

In this study, we used TAC as a model to induce HF due to pressure overload in mice to perform systemic analyses of fecal microbiota at different stages of HF (Figure 1A). Sham-operated mice were used as controls. We also maintained one cohort of mice that did not undergo any surgical procedure to identify whether the gut microbiome is impacted by thoracotomy-dependent stress, independent of TAC/Sham surgery. We earlier showed a state of compensated hypertrophy after 2 weeks, and the onset of decompensation and subsequent heart failure between 2 and 6 weeks after the TAC treatment [22]. Thus, fecal samples were collected on days 0, 7, 14, and 42, and were subsequently processed for total DNA extraction and 16S rDNA-sequencing-based microbiota assessment. Out of 258 samples (6,448,250 reads) processed using the DADA2 pipeline, 26 samples were excluded by applying a filtering cut-off of 10,000 total reads; hence, our final sequence table comprised 214 samples (TAC: *n* = 90, Sham: *n* = 52, control: *n* = 72) with an average number of 23,651 sequences with a standard deviation of 6636 per sample. These samples resulted in 3081 amplicon sequence variants (ASVs), out of which, 1585 ASVs with an overall abundance count of more than 10 were used for further taxonomic assignments (Supplementary Table S1).

To assess the alpha-diversity (a measure for intraindividual bacterial variance), we calculated the Shannon diversity index for both richness and evenness based on amplicon sequence variants count in mice that underwent TAC or a Sham procedure, as well as untreated controls. A significant decrease in alpha-diversity was observed in TAC operated mice compared to Sham controls on days 7, 14, and 42 (*p* = 0.031, *p* = 0.064, *p* = 0.004) (Figure 1B). In contrast, the Shannon diversity in Sham mice compared to control mice revealed no significant differences on days 0, 7, 14, and 42 (Supplementary Figure S1A). Interestingly, we observed a gradual progressive decrease in alpha-diversity on days 7, 14, and 42 (*p* = 0.014, *p* = 0.0016, *p* = 0.0021) compared to day 0, which coincided with compensated hypertrophy, maladaptive hypertrophy, and failing hearts, respectively (Figure 1B). Again, no significant differences were observed between fecal samples from Sham-operated mice and untreated controls at different time points (Figure 1C). Principal coordinate analysis (PCoA) based on GUniFrac distances in fecal samples from the control, Sham, and TAC samples at different time points showed distinct clustering on day 42 after the TAC treatment. Beta diversity analyses indicated that the effects were more pronounced at later time points of the experiment, which eventually led to a significant change in microbial composition (Figure 1D). Separate PCoA in TAC- and Sham-treated mice also showed distinct clustering on day 42 in the TAC group with more interindividual variations than the Sham group, demonstrating that time progression leads to profound changes in intestinal microbiota (Figure 1E,F). PCoA plots comparing TAC, Sham, and control mice at each time point separately showed no distinct grouping on day 0, indicating no significant baseline differences in the three groups before starting the experiment (Supplementary Figure S1B). However, on day 7, analysis of the TAC- and Sham-treated mice showed distinct clusters compared to the controls (Supplementary Figure S1C). On days 14 and 42, distinct clustering was observed only in fecal samples from TAC-treated mice, indicating more variations in diversity (Supplementary Figure S1D,E).

**Figure 2:**
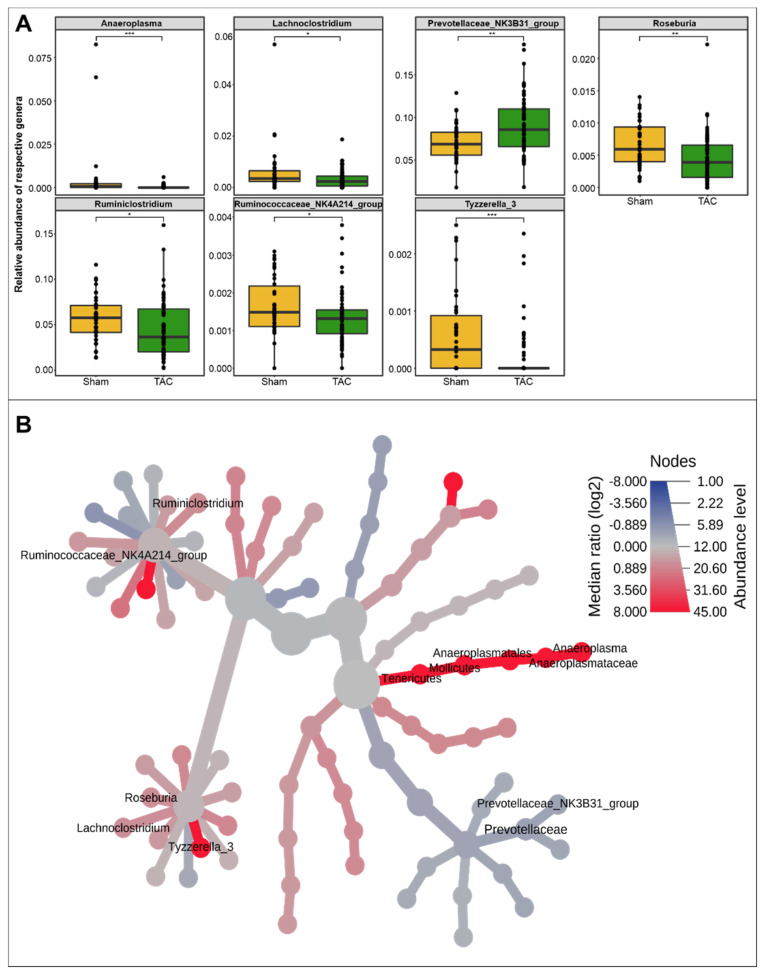
Comparative analysis of gut microbial content in Sham Vs. TAC mice. (**A**) Box plot showing relative abundances of the total of 7 genera that were found to be differentially abundant using Wilcoxon’s test in the Sham and TAC comparison, indicating that most of the genera were found to be more abundant in Sham samples as compared to TAC samples (*, **, and *** correspond to *p* ≤ 0.05, *p* ≤ 0.01, and *p* ≤ 0.001, respectively). (*n* values are as follows: Sham = 38, TAC = 63). (**B**) Heat-tree showing significantly different genera in the Sham and TAC comparison using Wilcoxon’s test (*p*-value ≤ 0.05). (*n* values are as follows: Sham = 38, TAC = 63).

Further dissection of the taxonomy at the genus level for fecal samples using relative abundance data in the TAC, Sham, and control mice for the top 20 genera showed that most of the variations were present in the TAC mice. The *Lachnospiraceae NK4A136* group from the Firmicutes phylum was found to be the most abundant genus in all groups, which gradually decreased with the progression to HF in TAC mice but remained unaltered in Sham and control groups (Supplementary Figure S2). Similarly, the abundance of *Mucispirillum*, *Roseburia*, and *Desulfovibrio* was reduced in TAC mice as compared to their initial abundances (Supplementary Figure S2). On the other hand, *Bacteroides*, *Prevotellaceae UCG-001*, *Alistipes*, *Parasutterella*, *Ruminococcaceae UCG-014*, and *Ruminococcus 1* were found to increase gradually with time in TAC mice. At the same time, there were only minor changes in their abundances in Sham and control samples (Supplementary Figure S2). Furthermore, the *Prevotellaceae NK3B31* group, *Ruminiclostridium*, *Roseburia*, *Anaeroplasma*, *Lachnoclostridium*, the *Ruminococcaceae NK4A214* group, and *Tyzzerella_3* genera were found to be significantly abundant in TAC and Sham mice, as calculated by Wilcoxon’s test (Figure 2A,B). *Tyzzerella_3*, *Roseburia*, and *Ruminiclostridium* were more abundant in Sham mice, while the *Prevotellaceae NK3B31* group genus abundance was higher in TAC mice (Figure 2A).

### 3.2. Severe HF Was Associated with Aggravated Alterations in Intestinal Microbiota Compared to Mild or Moderate HF

**Figure 3:**
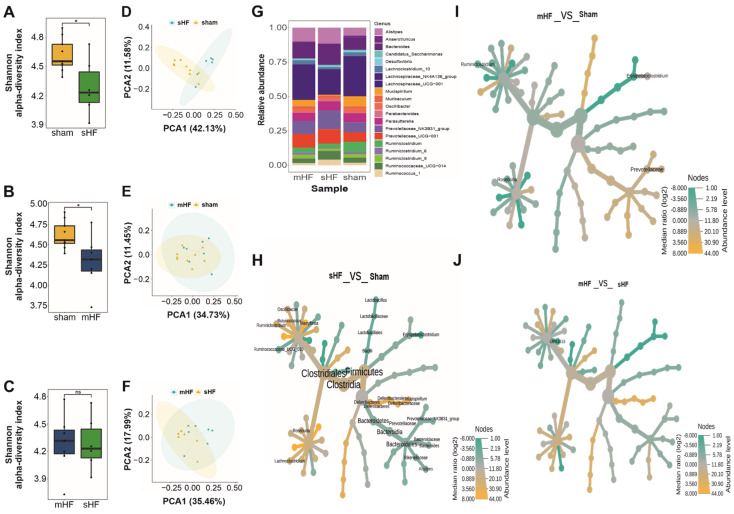
Diversity and microbial compositional differences in the mHF, sHF, and Sham samples. (**A**) Box plot for Shannon diversity index showing a significant difference in sHF vs. Sham samples (*p*-value = 0.038). (**B**) Box plot for the Shannon alpha-diversity index showing significant differences in the mHF and Sham samples (*p*-value = 0.039). (**C**) Box plot for the Shannon alpha-diversity index for the mHF and sHF samples showing no significant differences in samples. (**D**) PCoA plot with separate clustering in the sHF and Sham samples (PERMANOVA *p*-value: 0.258, beta dispersion *p*-value: 0.2498). (**E**) PCoA analysis of the mHF and Sham samples with no distinct cluster formation (PERMANOVA *p*-value: 0.411, beta dispersion *p*-value: 0.3976). (**F**) PCoA analysis results of the sHF and mHF samples showing no significant differences (PERMANOVA *p*-value: 0.733, beta dispersion *p*-value: 0.7322). (**G**) Top twenty genera relative abundances in the mHF, sHF, and Sham sample types. (**H**) Heat-tree showing 13 differentially abundant taxa in the sHF vs. Sham comparison using Wilcoxon’s test. (**I**) Heat-tree for significantly abundant taxa between the mHF and Sham samples comparison using Wilcoxon’s test. (**J**) Heat-tree showing no significant differences in the mHF and sHF samples using Wilcoxon’s test. (*n* values for 3F–3J are as follows: Sham = 9, mHF = 7, sHF = 6). * and ns correspond to *p* ≤ 0.05, *non-significant*, respectively.

To analyze the impact of the degree of HF on intestinal microbiota at day 42, we grouped the animals into two sub-groups (based on the echocardiography parameters at day 42): those that had an LVEF < 40% (severe HF, sHF, *n* = 8) after the TAC procedure and those that had an LVEF from 40–55% (mild-to-moderate HF, mHF, *n* = 9) (Supplementary Figure S3A–E). We observed no significant differences in the alpha-diversity of the mHF (mean = 4.29, SD = 0.32) and sHF (mean = 4.28, SD = 0.29) samples (Figure 3A–F). A comparison of the top 20 genera in the Sham, sHF, and mHF mice revealed that the *Prevotellaceae_NK3B31*_group and *Ruminococcaceae_UCG-014* genera were abundant in sHF. Meanwhile, the *Prevotellaceae_UCG-001*, *Ruminococcus_1*, *Alistipes*, *Ruminiclostridium_6*, and *Bacteroides* genera were abundant in both the sHF and mHF sub-groups compared to the Sham group. In contrast, the *Lachnospiraceae_NK4A136*_group, *Ruminiclostridium*, and *Mucispirillum* genera were less abundant on day 42 in mice that received the TAC procedure (both sHF and mHF) (Figure 3G).

Furthermore, the Firmicutes and Deferribacteres phyla levels were reduced by almost 50% in sHF compared to the Sham controls. Meanwhile, the abundance of genera from the Bacteroidetes phylum was exceptionally high in sHF, indicating a major shift in microbial composition in severe HF cases. A total of 13 genera were found to be differentially abundant in Sham versus sHF mice calculated using Wilcoxon’s test, out of which, *Mucispirillum*, *Oscillibacter*, *Ruminiclostridium*, *Roseburia*, *Butyricicoccus*, *Lachnoclostridium*, and *Harryflintia* were significantly reduced in the sHF as compared to Sham controls (Supplementary Figure S3F). Conversely, the *Prevotellaceae_NK3B31*_group, *Bacteroides*, *Alistipes*, *Lactobacillus*, *Ruminococcaceae_UCG-010*, and *Erysipelatoclostridium* accumulated in samples from mice with sHF (Figure 3H). When comparing bacteria in fecal samples from mice with mHF to Sham controls, a total of only three genera were found to be differentially abundant, as calculated using Wilcoxon’s test. *Ruminiclostridium* and *Roseburia* were significantly reduced, while *Erysipelatoclostridium* was highly abundant in mHF compared to the Sham group (Figure 3I). Interestingly, however, no significant differences were observed between mHF and sHF, except for the UBA1819 genus from the *Ruminococcaceae* family, which was significantly more abundant in sHF compared to mHF (Figure 3J).

### 3.3. Functional Metabolic Analysis of the Gut Microbiome Revealed Altered Carbohydrate, Lipid, and Amino Acid Metabolism in Failing Hearts

To predict the functional impact of altered gut microbial communities from 16S rRNA sequencing data, we carried out a KEGG orthology analysis using PICRUSt2. Functional comparison of fecal microbiota and metabolites in mice with sHF, mHF, and Sham controls at different KEGG annotation levels indicated significant differences in the abundances of imputed KEGG modules related to carbohydrate, lipid, and amino acid metabolism using the Kruskal–Wallis test (Table 1). Specifically, KEGG modules related to the microbial metabolites short-chain fatty acids (SCFAs), trimethylamine-N-oxide (TMAO), bile acid metabolism, and amino acid metabolism were significantly altered between these groups.

#### 3.3.1. Bile Acid Metabolism

**Figure 4:**
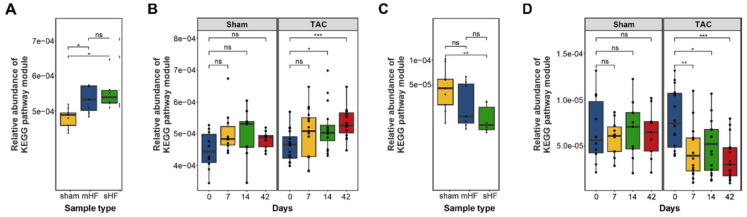
Abundances of KEGG pathway modules were found to be significant using Kruskal–Wallis multivariate analysis related to bile acid synthesis. (**A**) Relative abundance of the choloylglycine hydrolase gene in the sHF, mHF, and Sham samples (Kruskal–Wallis test *p*-value = 0.0037). (*n* values are as follows: Sham = 9, mHF = 7, sHF = 6). (**B**) Relative abundance of the choloylglycine hydrolase gene in the Sham and TAC samples at different time points showing an increase in the concentration of the gene with time in TAC samples as compared to Sham samples (Kruskal–Wallis test *p*-value = 0.23 for Sham samples and *p*-value = 0.01 for TAC samples). (*n* values are as follows: Sham day 0 = 10, day 7 = 10, day 14 = 9, day 42 = 9; TAC day 0 = 16, day 7 = 16, day 14 = 16, day 42 = 15). (**C**) Relative abundance of 7-α-hydroxysteroid dehydrogenase gene in the sHF, mHF, and Sham samples with a significant decrease in sHF and mHF compared to the Sham samples (Kruskal–Wallis test *p*-value = 0.042). (*n* values are as follows: Sham = 9, mHF = 7, sHF = 6). (**D**) Relative abundance of 7-α-hydroxysteroid dehydrogenase gene in the Sham and TAC samples at different time points with a significant decrease in abundance with time in the TAC samples (Kruskal–Wallis test *p*-value = 0.0025). (*n* values are as follows: Sham day 0 = 10, day 7 = 10, day 14 = 9, day 42 = 9; TAC day 0 = 16, day 7 = 16, day 14 = 16, day 42 = 15). *, **, and *** correspond to *p* ≤ 0.05, *p* ≤ 0.01, and *p* ≤ 0.001, respectively; ns, non-significant.

The bacterial enzyme choloylglycine hydrolase (bile salt hydrolase), which is required for the deconjugation step to remove glycine/taurine from conjugated primary bile acid, was found to be highly abundant in the imputed functional profile of the fecal samples of mice with sHF and mHF as compared to the Sham control mice (Figure 4A), which showed steady increases at days 7, 14, and 42 after the TAC treatment (Figure 4B). In contrast, 7-α-hydroxysteroid dehydrogenase, which is an essential enzyme for converting primary to secondary bile acid, was less abundant in the sHF and mHF mice than the Sham controls (Figure 4C) and progressively decreased at different time points after the TAC treatment (Figure 4D).

#### 3.3.2. Short-Chain Fatty Acids Metabolism

**Figure 5:**
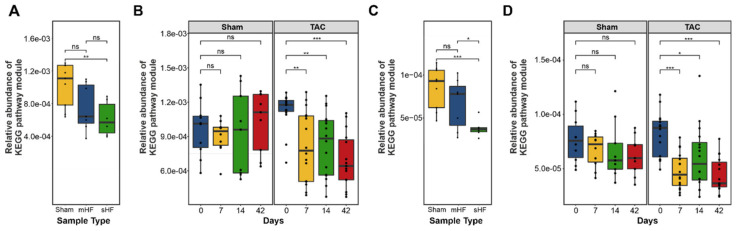
Box plot showing differences in the abundances of modules involved in butanoate and propanoate biosynthesis. (**A**) Differences in the abundances of genes involved in butanoate biosynthesis are shown for the sHF, mHF, and Sham samples. (*n* values are as follows: Sham = 9, mHF = 7, sHF = 6). (**B**) Genes involved in butanoate metabolism in the Sham and TAC samples at different time points indicating a significant decrease in gene concentrations in TAC samples with time. (*n* values are as follows: Sham day 0 = 10, day 7 = 10, day 14 = 9, day 42 = 9; TAC day 0 = 16, day 7 = 16, day 14 = 16, day 42 = 15). (**C**) Differences in the abundance of genes involved in propanoate biosynthesis are shown for the sHF, mHF, and Sham samples. (*n* values are as follows: Sham = 9, mHF = 7, sHF = 6). (**D**) Abundances of genes in propanoate biosynthesis at different time points in the Sham and TAC samples showing a decrease in gene concentrations in both the Sham and TAC samples with respect to time. TAC samples showed a significant decrease (*p*-value ≤ 0.05) for different time point samples relative to the initial (0th day) samples. (*n* values are as follows: Sham day 0 = 10, day 7 = 10, day 14 = 9, day 42 = 9; TAC day 0 = 16, day 7 = 16, day 14 = 16, day 42 = 15). *, **, and *** correspond to *p* ≤ 0.05, *p* ≤ 0.01, and *p* ≤ 0.001, respectively; ns, non-significant.

We also observed significant differences in butanoate and propanoate metabolism in the sHF, mHF, and Sham mice. A total of four predicted functional modules involved in propionate synthesis and six modules involved in butyrate synthesis showed less abundance in sHF and mHF than the Sham controls (Figure 5A–D). Furthermore, a total of 14 functional modules related to butyrate and propionate metabolism were significantly altered in the Sham and TAC samples at different time points (Supplementary Figure S4).

#### 3.3.3. TMA and TMAO Pathways

**Figure 6:**
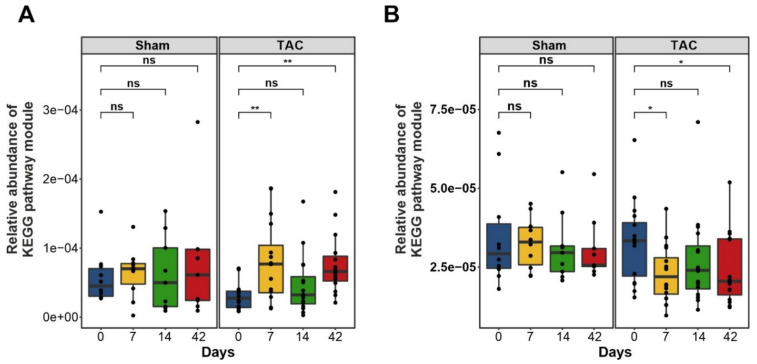
Box plot showing differences in the abundances of genes involved in the TMAO pathway in the Sham and TAC samples. (**A**) Differences in the abundances of the torZ gene are shown for the TAC and Sham samples. Significant differences in the torZ concentrations in TAC samples at the 1st and 6th weeks were found as compared to the initial TAC samples (0th week) using Wilcoxon’s test. (**B**) Differences in the abundances of the betaine/carnitine transporter involved in the TMAO pathway are shown for the TAC and Sham samples. The Wilcoxon’s test showed a significant decrease in gene concentration at the 1st and 6th weeks in the TAC samples. (*n* values are as follows: Sham day 0 = 10, day 7 = 10, day 14 = 9, day 42 = 9; TAC day 0 = 16, day 7 = 16, day 14 = 16, day 42 = 15). * and ** correspond to *p* ≤ 0.05, *p* ≤ 0.01 respectively; ns, non-significant.

The trimethylamine-N-oxide reductase (cytochrome c) (torZ) and betaine/carnitine transporter (BCCT) families involved in TMA synthesis using TMAO were less abundant in the Sham controls at different time points as compared to the TAC mice, which indicated why the TMAO levels were higher in mice after the TAC treatment (Figure 6A). Choline dehydrogenase (betA) (EC:1.1.99.1) (CHDH) and betaine-aldehyde dehydrogenase (betB, gbsA) (EC:1.2.1.8), which are required for the conversion of choline to betaine, and L-carnitine CoA-transferase (caiB) (EC:2.8.3.21), which is required for the conversion of L-carnitine to γ-butyrobetaine, were accrued more in the TAC samples as compared to the Sham samples (Figure 6B).

#### 3.3.4. Amino Acid Metabolism

**Figure 7:**
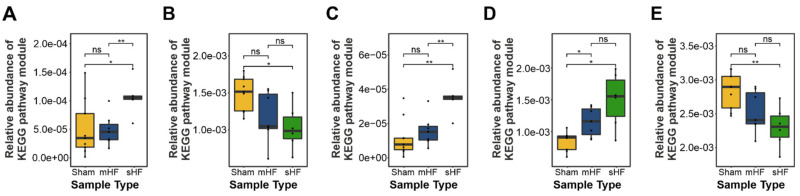
Box plots showing the differentially abundant KEGG pathway modules for amino acid metabolism. (**A**) Branched-chain amino acid degradation-related genes in the sHF, mHF, and Sham samples (Kruskal–Wallis test *p*-value = 0.001). (**B**) Aromatic amino acid metabolism in the sHF, mHF, and Sham samples (Kruskal–Wallis test *p*-value = 0.024). (**C**) Aromatic amino acid biosynthesis in the sHF, mHF, and Sham samples (*p*-value = 0.012). (**D**) Positively charged amino acid degradation in the sHF, mHF, and Sham samples (Kruskal–Wallis test *p*-value = 0.036). (**E**) Positively charged amino acid biosynthesis in the sHF, mHF, and Sham samples (Kruskal–Wallis test *p*-value = 0.018). (*n* values are as follows: Sham = 9, mHF = 7, sHF = 6). * and ** correspond to *p* ≤ 0.05, *p* ≤ 0.01 respectively; ns, non-significant.

Prominent differences in predicted KEGG modules related to amino acid metabolism were observed in the sHF, mHF, and Sham-operated mice. A significant increase in the genes involved in branched-chain amino acid degradation was observed in sHF mice compared to Sham and mHF animals (Figure 7A). Moreover, genes related to aromatic amino acid catabolism were significantly more abundant in the sHF group (Figure 7B). The essential amino acid tryptophan, which is the precursor of many physiologically essential metabolites, especially kynurenine, accounts for ~95% of the overall tryptophan degradation [36]. Metabolome data demonstrated an increase in the concentration of kynurenine as compared to tryptophan for week 6 in mice with sHF (Figure 7C). The degradation of positively charged amino acids was also significantly higher in mice with sHF followed by mice with mHF compared to Sham controls (Figure 7D), while the biosynthesis of positively charged amino acids was the lowest in mice with sHF followed by mHF as compared to Sham controls (Figure 7E).

### 3.4. Gut Dysbiosis Caused Alterations in Circulating Metabolites

**Figure 8:**
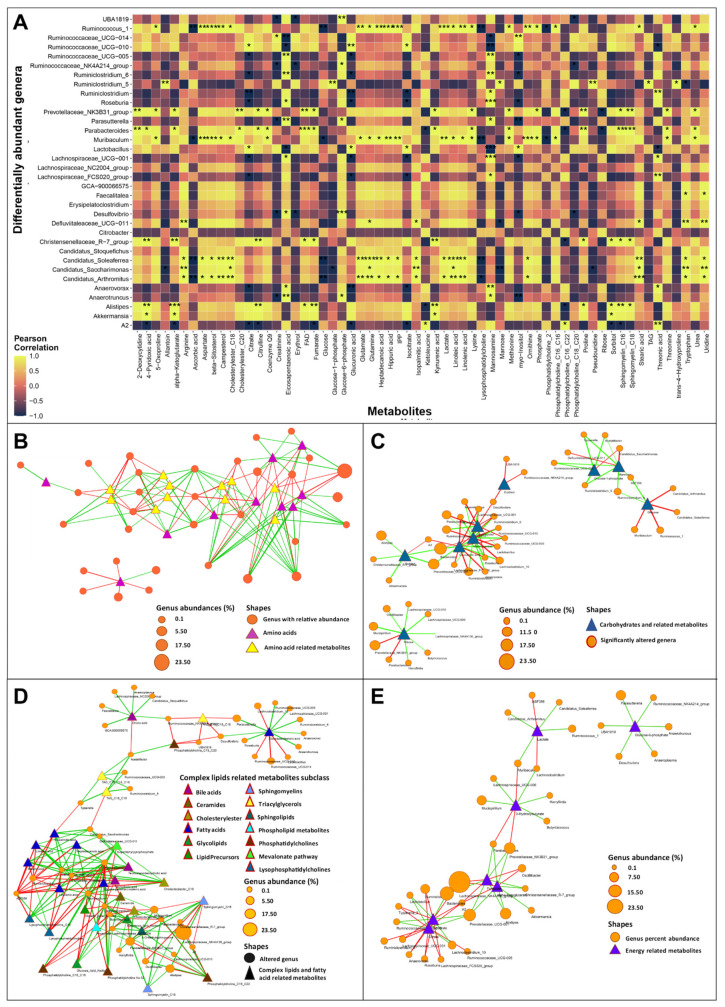
Serum metabolites and gut–microbiome correlation analysis. (**A**) Heatmap showing Pearson correlations between differentially abundant genera and metabolites with large differences in their ratios in the Sham and TAC samples with significance (* *p*-values ≤ 0.5, ** *p*-value ≤ 0.01, *** *p*-values ≤ 0.001). (**B**) Pearson correlation network in amino-acids-related metabolites and differentially abundant genera in the Sham and TAC samples. (**C**) correlation network between carbohydrate-related metabolites and differentially abundant genera. (**D**) correlation network between complex lipids/fatty acids and differentially abundant genera. (**E**) correlation network between energy-metabolism-related metabolites and differentially abundant genera. (*n* values are as follows: Sham = 38, TAC = 63).

To advance the understanding of the role, if any, played by gut bacterial dysbiosis in metabolic changes in HF, Pearson’s correlation analysis was conducted to compare 50 altered genera (genera with ≥0.01% abundance) with 81 metabolites involved in amino acid metabolism, energy metabolism, complex lipids and fatty acid metabolism, along with the vitamins and related metabolites and nucleobases and related metabolites classes. For metabolites, an ANOVA test was carried out on ratios of the TAC and Sham samples at 14 and 42 days, and molecules with significant *p*-values or showing large differences in their ratios (≥0.3 or ≤−0.3) were considered for correlation calculations (Figure 8A).

A total of 21 amino acids and related metabolites were found to be significantly different in the TAC and Sham samples at different time points. ASV contributions to KEGG modules and correlation analysis indicated that most of the amino acids and their synthesis-related KEGG functional modules (tryptophan, tyrosine, arginine, and lysine biosynthesis) were positively associated with the genera *Candidatus Saccharimonas*, *Candidatus Arthromitus*, *Candidatus Soleaferrea*, *Defluviitaleaceae UCG-011*, *Muribaculum*, *Prevotellaceae NK3B31* group, and *Ruminococcus_1*. At the same time, *ASF356*, *Lachnoclostridium*, *Tyzzerella*, *Lachnospiraceae_UCG-006*, and *Ruminiclostridium_9* were negatively correlated with specified KEGG modules (Figure 8B). Of note, tryptophan catabolism leads to kynurenic acid formation, which is known to be associated with several cardiovascular disease entities, including HF [37,38]. Consistently, we found a significant increase in kynurenic acid in TAC samples at different time points. Furthermore, the KEGG modules K00453 (TDO2 kynA; tryptophan 2 3-dioxygenase (EC:1.13.11.11)) and K01556 (KYNU kynU; kynureninase (EC:3.7.1.3)), which are essential enzymes for the formation of kynurenic acid from tryptophan, were significantly increased in TAC samples as compared to the Sham controls over time. These modules might have been contributed to by ASVs from the genera *Propioniciclava* and *Diaphorobacter* and the family *Burkholderiaceae*, which were more abundant in the TAC samples than the Sham controls (Figure 8B). Tryptophan catabolism also leads to the formation of 3-indoxy sulfate, which is a uremic toxin that was found to be correlated with adverse cardiovascular events [39]. We observed an increased concentration of 3-indoxy sulfate in TAC mice and a strong positive correlation with the *Alistipes* genus (Figure 8B). ASVs belonging to the *Alistipes* genus were predicted to express the tnaA; tryptophanase (EC:4.1.99.1) enzyme by PICRUSt2, likely explaining the strong correlation between the *Alistipes* genus and 3-indoxy sulfate. Creatinine, which is another compound derived from amino acid catabolism related to cardiovascular disease, was also comparatively higher in the TAC samples. It was positively correlated with *Ruminococcaceae_UCG-014* and negatively correlated with *Parasutterella*, *Desulfovibrio*, *Anaerotruncus*, *UBA1819*, and *Ruminococcaceae_NK4A214*_group (Figure 8B).

Changes in microbiota composition affect carbohydrate metabolism, e.g., intestinal microbial alterations, have been linked to changes in insulin sensitivity and glucose metabolism [40]. Glucose concentration was found to be decreased in TAC samples with time, while it remained unchanged in the Sham samples. These observations could be positively correlated to *Ruminiclostridium_9* and *ASF356* genera, and negatively correlated to *Ruminococcus_1*, *Muribaculum*, *Candidatus Arthromitus*, and *Candidatus Soleaferrea* (Figure 8C).

Primary bile acid (cholic acid, taurocholic acid, and taurochenodeoxycholic acid) levels were increased in the blood of the TAC mice, which was negatively correlated with the abundance of *Ruminiclostridium_9*, *Acetatifactor*, *ASF356*, and *Lachnoclostridium* (Figure 8D). On the other hand, the circulating levels of ceramide were reduced in the TAC mice and were positively correlated with the *Lachnospiraceae_UCG-006*, *Butyricicoccus*, and the *Lachnoclostridium* genera from the phylum firmicutes and negatively correlated with the *Ruminococcus_1* and *Muribaculum* genera (Figure 8D).

Recent studies indicated an increase in myocardial utilization of 3-hydroxybutyrate (3-OHB) in patients with HF [41]. Along these lines, we found a decrease in the serum concentration of 3-OHB in the TAC mice with time compared to the Sham-operated mice. 3-OHB was further positively correlated with *Mucispirillum*, *Lachnospiraceae_UCG-006*, *Butyricicoccus*, *Lachnoclostridium*, and *Harryflintia*, and negatively correlated with the *Prevotellaceae_NK3B31*_group, *Muribaculum*, and *Parabacteroides* genera (Figure 8E).

## 4. Discussion

We and others have previously demonstrated gut microbiome alterations in HF patients [7,9,10]. To further assess the direct effects of HF on gut dysbiosis in a controlled and progressive setup, here we analyzed the time-dependent changes in intestinal microbiota using fecal samples from mice that developed HF of different severity in response to pressure overload. Extending the knowledge of HF–gut dysbiosis associations from recent findings [42,43], we observed that HF led to a steady decrease in the alpha-diversity of intestinal bacterial communities over time compared to control mice, pointing toward the notion that sustained HF leads to progressive changes in intestinal microbiota. Furthermore, the alterations were closely associated with the severity of the HF condition.

Butyrate, which is a microbiota-derived SCFA, the levels of which are known to be reduced in human HF patients [8], was shown to attenuate inflammation and myocardial hypertrophy and to improve cardiac function following myocardial infarction [44,45]. Interestingly, for the first time in mice, we observed a significant decrease in butyrate and propanoate synthesis and metabolism in mice with HF compared to Sham controls. In our HF study, *Lachnospiraceae_NK4A136*_group was the most abundant genus in all groups at the initial time point, which gradually decreased with time in the TAC group but remained unchanged in the Sham and control groups. Kamo et al. found a reduced relative abundance of *Eubacterium rectale* and *Dorea longicatena* (Lachnospiracea family) and levels of *Faecalibacterium* (*Ruminococcaceae* family) were lower in older patients [9]. In a metagenomic analysis, Cui et al. demonstrated reduced levels of *Faecalibacterium prausnitzii* in patients with HF [8]. A common finding in these studies was the relative reduction in taxa from the *Lachnospiracea* or *Ruminococcacea* families, which are known for their capacity for butyrate production. *Lachnospiraceae_NK4A136*_group is a butyrate-producing bacteria that was recently shown to exhibit anti-inflammatory effects and proposed to have a probiotic potential [46,47] (DOI: https://doi.org/10.21203/rs.3.rs-48913/v1, accessed on 24 August 2021). Future research could thus be directed toward exploring the beneficial effects, if any, of maintaining the gut levels of *Lachnospiraceae_NK4A136*_group, e.g., through probiotic administration or butyrate supplementation.

Similarly, *Lactobacillus* spp., which is a well-established lactic-acid-producing probiotic bacteria that also carry anti-inflammatory and cholesterol-lowering effects [48,49], were significantly reduced in mice with severe cardiac dysfunction. In a recent report using the TAC mouse model in C57BL/6J mice, ferulic acid increased intestinal *Lactobacillus* abundance and improved cardiac function [50]. Furthermore, *Lactobacillus acidophilus*, *Lactobacillus reuteri*, and several strains of *Lactobacillus plantarum* have cardioprotective effects mitigating doxorubicin-induced cardiomyopathy in rats [48,49,51]. Interestingly, a recent report illustrated that a probiotic mixture of *Lactobacillus plantarum* strains not only improve metabolic syndrome (one of the hallmarks of HF with preserved ejection fraction (HFpEF)) but also enriched *Lachnospiraceae_NK4A136*_group [46]. These findings suggested that the use of *Lactobacillus plantarum* strains might serve a dual role when protecting the heart, first by exerting beneficial effects of its own, and second, by enriching other likely beneficial bacteria, e.g., *Lachnospiraceae_NK4A136*_group.

Intriguingly, we also observed differential regulation of specific amino acids and their biosynthesis precursors between the TAC and Sham mice in close association with intestinal dysbiosis. For example, tryptophan, tyrosine, arginine, and lysine biosynthesis was found to be positively associated with the genera *Candidatus Saccharimonas*, *Candidatus Arthromitus*, *Candidatus Soleaferrea*, *Defluviitaleaceae UCG-011*, *Muribaculum*, *Prevotellaceae NK3B31* group, and *Ruminococcus_1*, while *ASF356*, *Lachnoclostridium*, *Tyzzerella*, *Lachnospiraceae_UCG-006*, and *Ruminiclostridium_9* were negatively correlated with the specified KEGG modules. Tryptophan catabolism leads to kynurenic acid formation, which is known to be associated with several cardiovascular diseases, including HF [52]. Furthermore, elevated plasma levels of metabolites of the kynurenine pathway and metabolite ratios are associated with increased mortality in patients with HF [38]. We also found a significant increase in the ratio of kynurenic acid in TAC samples at different time points. Furthermore, tryptophan catabolism also leads to the formation of 3-indoxy sulfate, which is a uremic toxin that is also shown to be correlated with adverse cardiovascular events [53]. ASVs belonging to the *Alistipes* genus predicted to express the tnaA tryptophanase enzyme showed a strong correlation of the *Alistipes* genus and 3-indoxy sulfate. This finding points to a possible mechanism contributing to the generation of harmful metabolites in mice with HF.

Altogether, the results of our study demonstrate a direct correlation of disruption of intestinal microbiota with the severity of HF. Our study was the first to precisely describe the simultaneous changes in intestinal microbiota and metabolome in a mouse model of pressure overload and demonstrated that intestinal microbiota and the associated metabolites were significantly altered in mice with HF. These findings are the first line of evidence showing the direct impact of HF on gut dysbiosis and associated microbial metabolites. Findings from the present study in conjunction with other reports would be valuable for the future course of research, where researchers will now have to establish whether altering the gut microbiome would impact cardiac function.

With advancements in sequencing technologies and an increasing number of focused studies identifying the gut microbial association with HF, the gut microbiome is looked to as a novel therapeutic target for the treatment of cardiovascular disease [54], and potential strategies for targeting intestinal microbial processes need to be taken. Steps are being taken in these directions, e.g., GutHeart human trial, which is a Phase II human trial that aimed to improve cardiac function in HF patients using antibiotics or probiotic yeast *Saccharomyces boulardii* to target the gut microbiome [55]. However, 3 months’ treatment with *S. boulardii* or rifaximin did not significantly affect LVEF, microbial diversity, or the measured biomarkers in a study population including HF patients [56]. Notwithstanding, we believe that these are important steps forward with gut-microbiome-targeted heart failure therapy and long-term interventions might exhibit significant results.

**Limitations**: Although only 85% of genes are conserved between mice and humans [57], the mouse is by far the most important animal model for human disease. Given that intestinal microbes can both colonize the mouse and human gut in principle, microbiome research could potentially overcome the species barrier between humans and mice. In this regard, “humanizing” mice with human microbiota seemed quite successful since 88% of genus-level taxa were found both in mice and donor samples [58]. However, it was already demonstrated that there are significant differences in the gut microbiota between healthy humans and WT mice [59]. Of note, several apparent differences between the intestinal tract of mice and humans received considerable attention, e.g., as diet has a strong effect on the microbiome composition of the intestine in humans [60]. Moreover, there are differences in gut pH, emptying rate of the gut, etc. [61]. Nevertheless, we believe that mouse models are adequate systems to study the impact of the microbiome on specific diseases, such as HF, because the pivotal role of the gut on the immune system and other pathophysiologic conditions appears to be conserved between different species [61]. Moreover, the mouse model allows for the standardization of food intake and other environmental factors such that the microbiome may be the only variable in a given experiment. Eventually, the key to a better understanding of the role of the microbiome in cardiovascular health and disease might be a focus on reproducing microbial differences at different locations with different mouse strains to truly show a robust effect of the relative contribution of diet, genotype, and environmental factors on the microbial composition. We believe that our study can deliver an important contribution to this canon.

## 5. Conclusions

In conclusion, the results of our study indicated that severe HF, as opposed to mild-to-moderate HF, was associated with marked dysbiosis. Overall, changes that were already observed in mice with moderate HF were more pronounced in mice with severe HF. This supports the hypothesis that HF is the underlying pathology of the disruption of microbiota and that the degree of HF correlated with the changes in intestinal microbiota. We hypothesized that the marked changes in the microbiome observed in severe HF further worsen cardiac function by reciprocating directly, e.g., via bacterial toxins, or indirectly, e.g., via metabolites or their intermediates. Our study comprehensively described the alteration of intestinal microbiota during HF in a standardized model. We identified distinct bacterial populations and the associated metabolites that are altered, and thereby provide a basis for future interventional studies, e.g., by probiotics or supplementation/suppression of specific metabolites.

## Figures and Tables

**Table 1:** Level 2 KEGG annotations using multivariate analysis in fecal samples of mice with sHF versus mHF versus Sham and Sham versus TAC mice (* and ** correspond to *p* ≤ 0.05 and *p* ≤ 0.01, respectively, ns: non-significant).

BRITE Hierarchy Pathway Module (Level 2)	*p*-Value—mHF vs. sHF vs. Sham	*p*-Value—Sham vs. TAC
Environmental adaptation	**	*
Protein families: metabolism	**	ns
Biosynthesis of other secondary metabolites	*	ns
Cellular community—prokaryotes	*	*
Unclassified: genetic information processing	*	ns
Cell growth and death	*	ns
Digestive system	*	ns
Metabolism of terpenoids and polyketides	*	ns
Transcription	*	*
Carbohydrate metabolism	*	*
Membrane transport	*	*
Metabolism of other amino acids	*	ns
Unclassified: metabolism	*	*
Nucleotide metabolism	*	ns
Lipid metabolism	*	ns
Glycan biosynthesis and metabolism	*	ns
Signal transduction	*	ns
Protein families: signaling and cellular processes	*	ns
Cell motility	*	ns
Energy metabolism	*	ns
Protein families: genetic information processing	*	ns
Unclassified: signaling and cellular processes	*	ns

## Data Availability

The 16S rDNA-sequencing data was deposited to the European Nucleotide Archive (ENA, https://www.ebi.ac.uk/ena/browser/home, accessed on 25 February 2022) and is available under the accession number PRJEB45533.

## Supplementary Materials

The following supporting information can be downloaded at https://www.mdpi.com/article/10.3390/biomedicines10040809/s1, Figure S1: Alpha and Beta diversity; Figure S2: Heatmap of relative abundance of top-20 genera at different time-points in TAC, sham and control samples; Figure S3: Echocardiography parameters and relative abundance of significantly abundant genera; Figure S4: Box plots showing differentially abundant KEGG pathway modules for amino acid metabolism in sham and TAC samples at different time-points; Table S1: amplicon sequence variants (ASVs) derived from DADA2.
