# Supplementary material for: Heart Failure Severity Closely Correlates with Intestinal Dysbiosis and Subsequent Metabolomic Alterations
Source: Biomedicines. 2022 Mar 30;10(4):809. doi: 10.3390/biomedicines10040809 (PMC9033061; doi:10.3390/biomedicines10040809)
Supplement: Supplementary file 1 [file biomedicines-10-00809-s001.zip › Gut microbiome Supplementary data_25.02.2022.pdf]

Supplementary Data

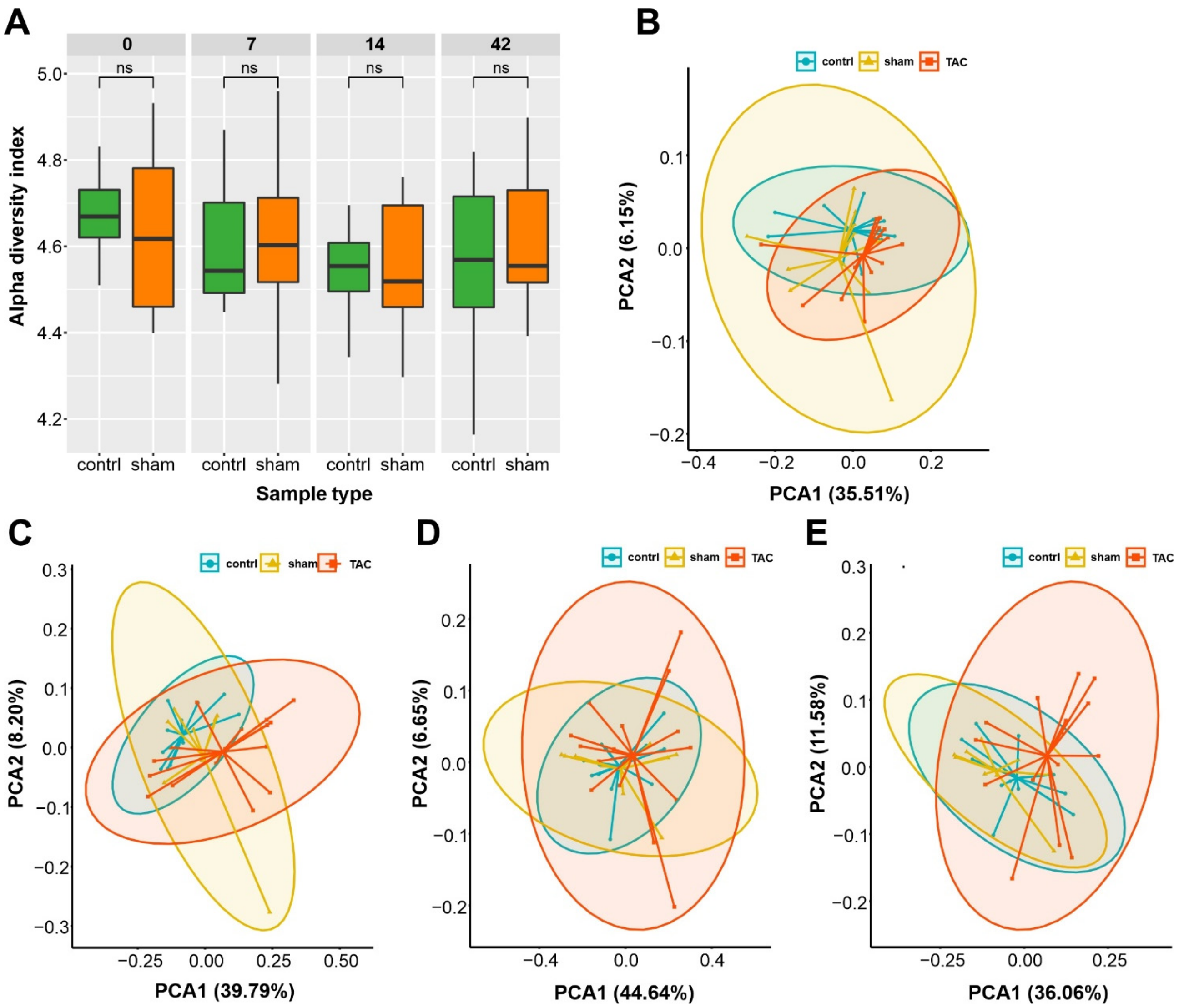

Supplementary Figure S1: Alpha and Beta diversity - **A:** Shannon alpha diversity index for sham and control sample-types with no significant differences at different time-points. **B:** PCoA plot showing for 0th day control, sham and TAC samples. **C:** PCoA plot showing significant difference in TAC and sham sample-types for 1st week (7th day). **D:** PCoA plot for 2nd week (14th day) sample comparison showing distinct cluster of TAC samples. **E:** PCoA plot for 6th week (42nd day) showing distinct cluster as compared to sham and control sample-types.

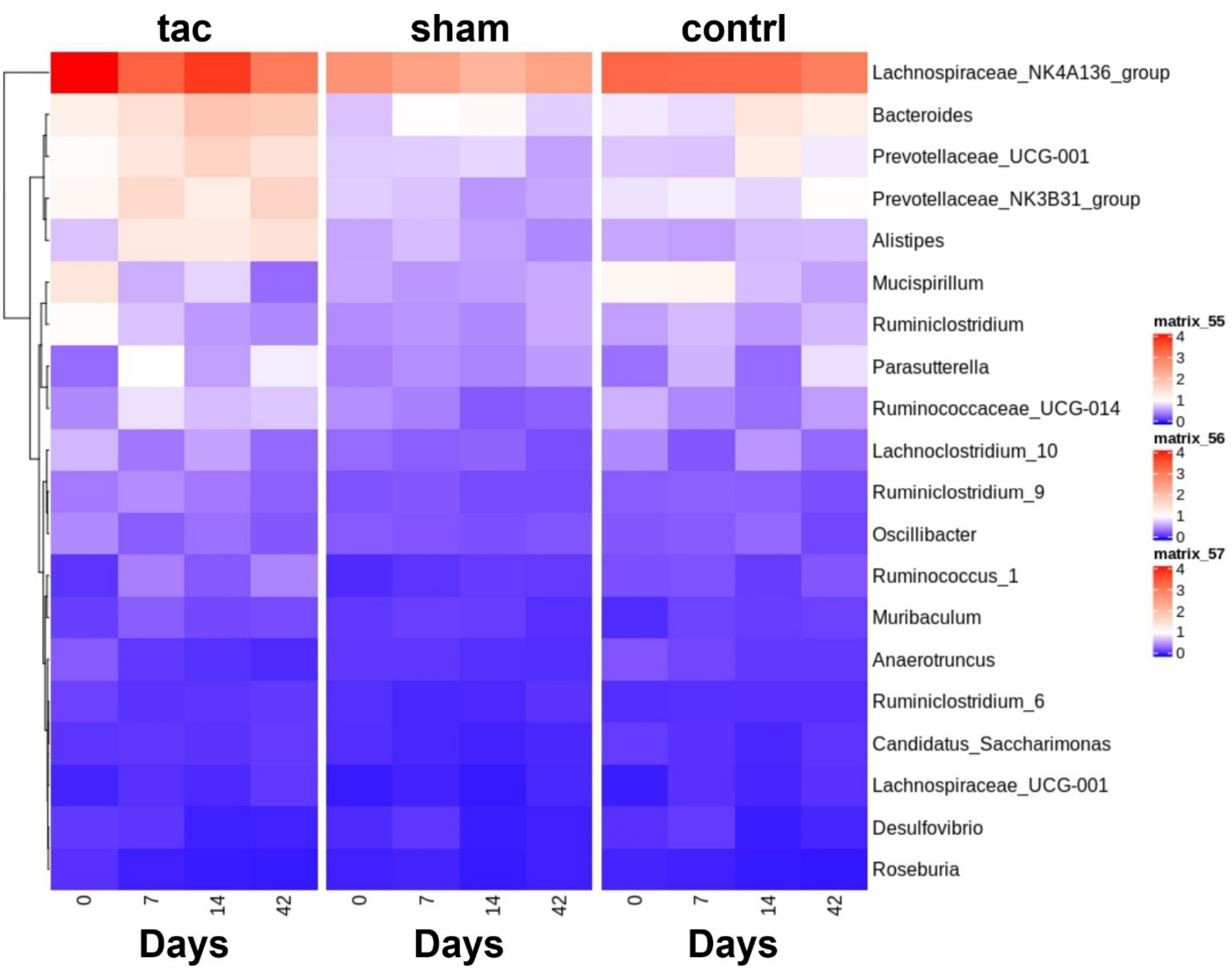

Supplementary Figure S2: Heatmap of relative abundance of top-20 genera at different time-points in TAC, sham and control samples. Variation in abundances of genera with different time-points were more in TAC samples as compared to sham and control samples.

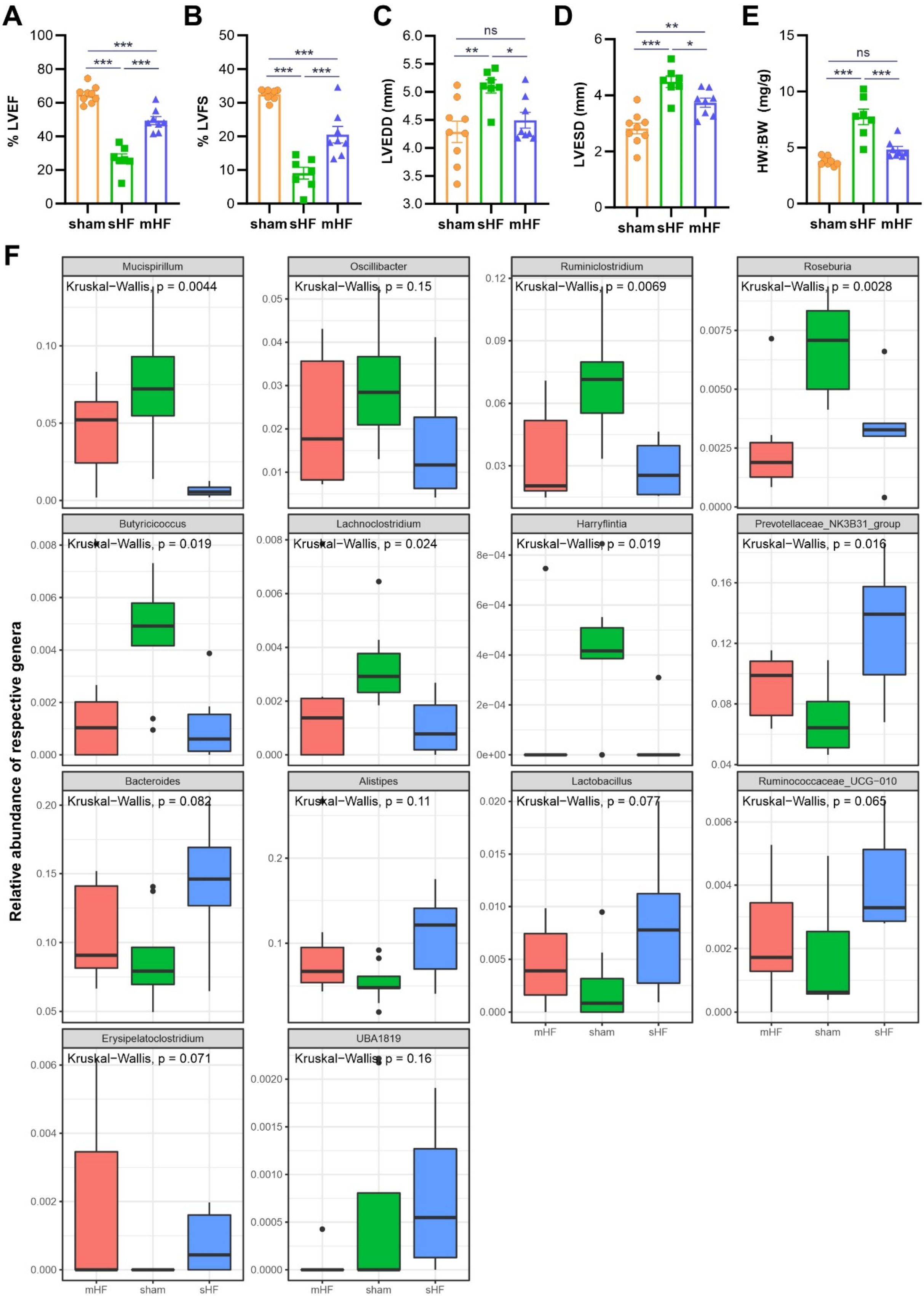

Supplementary Figure S3: Echocardiography parameters represented as bar graphs indicate severely reduced ejection fraction (**A**) and fractional shortening (**B**), and increased left ventricular end-diastolic- (**C**) and systolic- (**D**) diameters in sHF compared to mHF and sham groups. **E:** Bar graph showing heart weight to body weight ratios. (N for 3A - 3E = Sham – 9; mHF – 8; sHF - 7). **F:** Relative abundance of significantly abundant genera

found by Wilcoxon test *(P-value ≤ 0.05)* in sham, mHF and sHF samples. Total 13 genera with 3 phyla were found to be differentially abundant in sHF, mHF and sham comparisons.

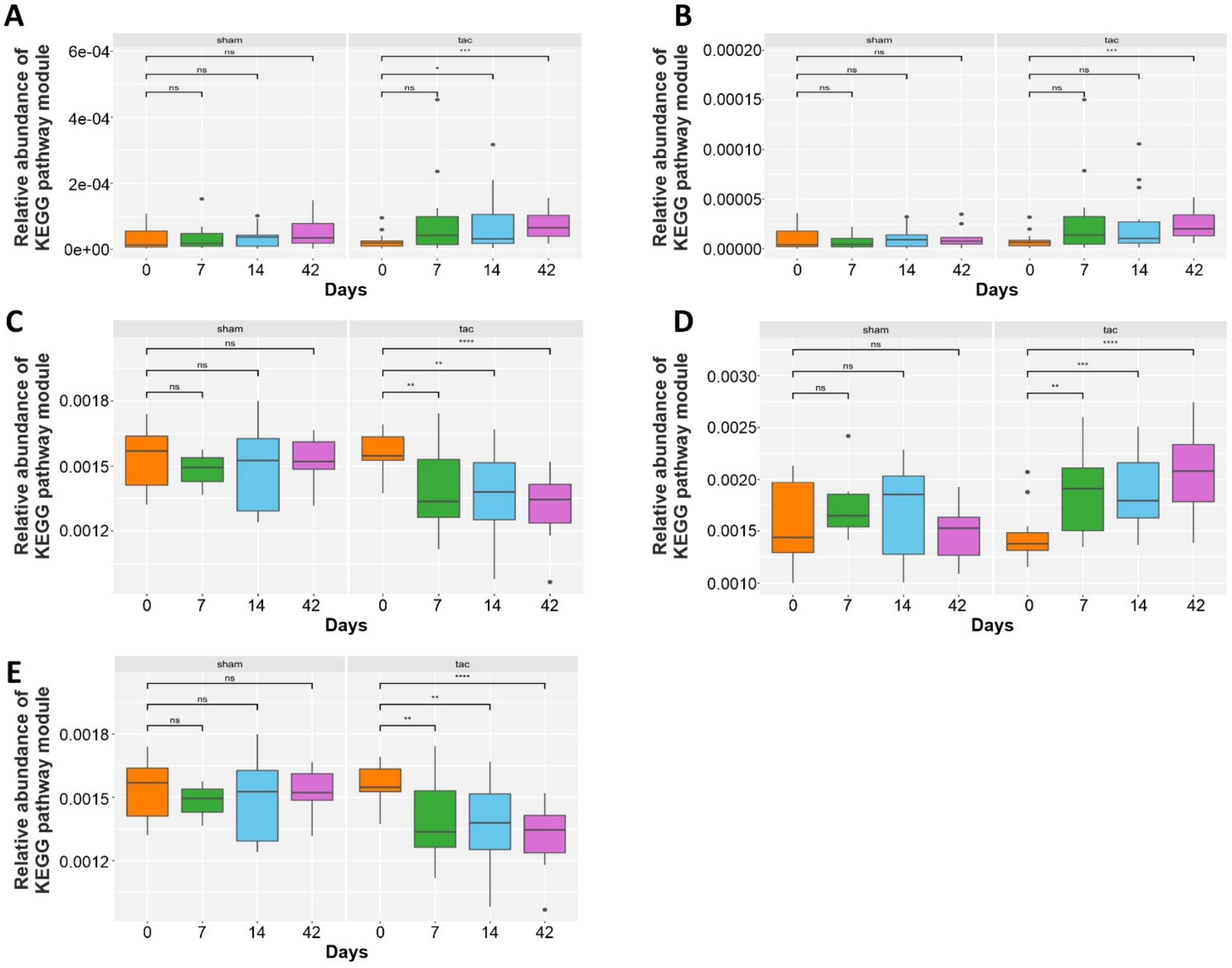

Supplementary Figure S4: Box plots showing differentially abundant KEGG pathway modules for amino acid metabolism in sham and TAC samples at different time-points. **A:** Branched chain amino acid degradation related genes in sham and TAC samples. Significant increase in BCAA degradation genes in TAC at 2nd and 6th week were found by Wilcoxon test. No significant differences observed in genes at different time-points in sham sanples. **B:** Aromatic amino acid metabolism in sham and TAC samples showing increase in genes at different time-points in TAC samples, with significant differences at 6th week. **C:** Aromatic amino acid biosynthesis related genes in sham and TAC samples at different time-points. Significant changes in abundances were observed in 6th week samples as compared to initial abundances of genes. **D:** Positively-charged amino acid degradation genes were found to be increased in TAC samples with respect to time significantly as compared to sham samples. **E:** Positively charged amino acid biosynthesis genes were found to be decreased in TAC samples by Wilcoxon test (p-value ≤ 0.05) for each time-point comparison with initial samples.
